# Diagnosis and analysis of primary central nervous system lymphoma based on MRI segmentation algorithm

**DOI:** 10.12669/pjms.37.6-WIT.4843

**Published:** 2021

**Authors:** Guanping Lu, Ying Li, Xinqiang Liang, Zhengjun Zhao

**Affiliations:** 1 Guanping Lu, Master of Medicine. Department of Neurosurgery, Nanxishan Hospital of Guangxi Zhuang Autonomous Region, Guilin City 541002, China; 2 Ying Li, Master of Medicine. Department of Neurosurgery, Nanxishan Hospital of Guangxi Zhuang Autonomous Region, Guilin City 541002, China; 3 Xinqiang Liang, Master of Medicine. Department of Neurosurgery, Nanxishan Hospital of Guangxi Zhuang Autonomous Region, Guilin City 541002, China; 4 Zhengjun Zhao, Bachelor’s Degrees. Department of Neurosurgery, Nanxishan Hospital of Guangxi Zhuang Autonomous Region, Guilin City 541002, China

**Keywords:** Primary central nervous system lymphoma, MRI, Diffusion-weighted imaging, Posterior cranial fossa, Magnetic resonance spectroscopy

## Abstract

**Objective:**

This paper summarizes the MRI imaging findings of primary central nervous system lymphoma (PCNSL) in the posterior cranial fossa to improve the accuracy of PCNSL diagnosis in the posterior cranial fossa.

**Methods:**

This study retrospectively analyzed the MRI imaging manifestations of 15 PCNSL posterior cranial fossa cases confirmed by puncture or surgical pathology from June 2017 to May 2018, including their occurrence sites, the number of lesions, MRI plain and enhanced manifestations, and diffusion-weighted imaging (DWI) and magnetic resonance spectroscopy. Imaging (MRS) performance.

**Results:**

A total of 15 cases were enrolled, including 10 cases of single lesion and five cases of multiple lesions. The total number of lesions was 25, which were in the cerebellar hemisphere and cerebellar vermis, midbrain, fourth ventricle, and pontine cerebellum. The lesions were round, irregular, nodular, patchy, with low or medium signals on T1WI, equal or slightly higher signals on T2WI, and enhanced with 25 meningiomas-like gray matter signals. All of them were significantly strengthened. “Acupoint sign” and “umbilical depression sign” were seen in eight lesions. There were 17 massive and nodular enhancements, four striped enhancements, three patchy enhancements, and one circular enhancement. five cases of DWI showed homogeneous high signal, two cases showed uneven high signal, and 3 cases showed medium signal. The ADC value of tumor parenchyma in 10 patients was (0.62±0.095)×10^-3^mm^2^/s. MRS examination showed obvious Lip peak in two cases.

**Conclusion:**

PCNSL in posterior cranial fossa has certain characteristics. DWI, ADC value and MRS are helpful to improve the correct diagnosis rate of PCNSL.

## INTRODUCTION

Primary central nervous system lymphoma (PCNSL) refers to non-Hodgkin’s lymphoma that only occurs in the central nervous system and no tumors are found elsewhere in the body.^1^ PCNSL has previously been reported in people with immunodeficiency or immune dysfunction, such as those with acquired immunodeficiency (such as AIDS, EB virus infection) or patients taking immunosuppressants for a long time.^2^ PCNSL is more common on the screen, it is relatively rare in the posterior cranial fossa, and the clinical manifestations are more atypical, and the misdiagnosis rate is higher.^3^ This study retrospectively analyzes the imaging data of 15 cases of PCNSL in the posterior cranial fossa confirmed by surgery or puncture pathology, and summarizes the MRI imaging manifestations to improve the accuracy of PCNSL diagnosis in the posterior cranial fossa.^4^

## TALLIES ENTROPY THRESHOLD SEGMENTATION

Tallies entropy, also called non-extended entropy, is an extension of Shannon entropy.^5^ The form of Tallies entropy is expressed as


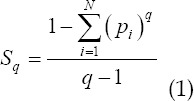


Among them: q is the undetermined coefficient, which describes the non-extensiveness of its entropy value; *p_i_* is the gray probability. Let the infrared image to be processed have N gray levels, and the histogram of the probability distribution of each gray level is represented as {*p_1_,p_2_,...,p_N_*}. Take the threshold t to divide the infrared image into two parts: target and background. The target probability is 
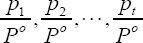
 and the background probability is 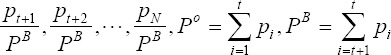. According to equation (1), the Tallies entropy of the target and background are obtained as


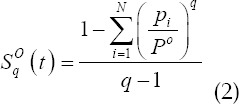



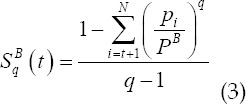


The magnitude of Tallies entropy changes according to the value of the threshold t. The total entropy value of the background and the target is


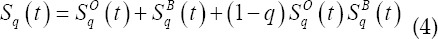


The process of finding the optimal threshold t to solve for the maximum value of *S_q_(t)*), that is,


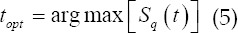


Traditional one-dimensional Tallies entropy threshold segmentation is susceptible to noise interference, especially for infrared images with low signal to noise.^6^ Therefore, using the two-dimensional Tallies entropy threshold segmentation method can reduce the influence of noise on the segmentation result.

According to the probability values of the target pixel and the background pixel, the Tallies entropy values of the target and background are obtained as


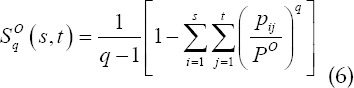



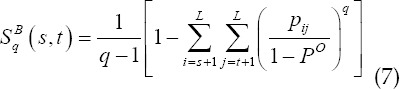


In summary, the two-dimensional Tallies entropy is


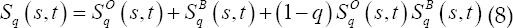


## METHODS

The imaging data of 15 patients with posterior cranial fossa PCNSL confirmed by pathology from June 2017 to May 2018 were collected in our hospital. There were 11 males and 4 females, aged 6 to 78 years, with an average age of (49.8±7.2) years. All patients had no history of congenital or acquired immune dysfunction, and had not been treated with hormone therapy before MRI, and secondary lymphoma was excluded by bone marrow cytology or other imaging tests (PET-CT). Fifteen patients were confirmed as PCNSL by surgery or stereotactic biopsy.

US GE750 3.0T superconducting magnetic resonance scanner and head and neck combined 8-channel coil was used. The scan sequences included T1WI, T2WI, and enhanced T1WI in axial, coronal, and sagittal positions. Ten patients underwent diffusion-weighted imaging (DWI) and four underwent magnetic resonance spectroscopy (MRS). The layer thickness is 6mm, the layer spacing is 1mm, the field of view (FOV) is 230mm×230mm, and the matrix is 384×384. T2WI: TR6000ms, TE90ms; T1WI: TR200ms, TE2.5ms; FLAIR: TR3500ms, TE90ms; DWI: TR3100ms, TE80ms. The enhanced scan uses a TSE sequence, the injection contrast agent is Gd-DTPA (0.1 mmol/Kg body weight), and the injection flow rate is 2 mL/s. Scan parameters: TR200ms, TE2.5ms, layer thickness 1mm, layer spacing 0. The DWI scan uses an EPI sequence, with b values of 0 and 1000s/mm2, TR3200ms, TE80ms, and FOV 230mm×230mm. MRS examination uses multi-voxel scanning, TR1600ms, TE125ms, FOV230mm×230mm, and the acquisition time is 8min. MRS post-processing was performed on the workstation to obtain the distribution map of each metabolite.

### Image analysis and statistical analysis

Two senior MRI physicians independently analyzed and measured the images, and observed the number, size, morphology, signal, and peritumoral edema of the lesions. When the opinions were not consistent, the two physicians discussed together. The statistical analysis software 20.0 was used to perform the Mann-Whitney U test on the ADC values of the solid part of the tumor and the contralateral brain parenchyma. P <0.05 was considered statistically significant.

## RESULTS

### Clinical manifestations

The manifestations included 11 cases of headache, 12 cases of dizziness, eight cases of malignant vomiting, six cases of unstable walking and ataxia. Only one case was diagnosed as lymphoma before operation, five cases were misdiagnosed as metastases, four cases were gliomas, one case was medulloblastoma, one case was inflammatory lesions, and three cases could not be characterized. Of the 15 cases, 12 were diffuse large B-cell lymphomas and three were Burkitt lymphomas.

### MRI findings

**Fig.1:**
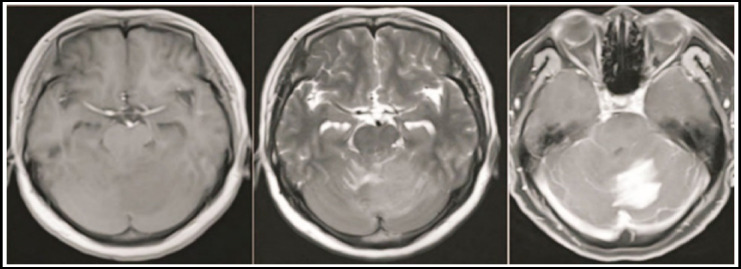
MRI image of diffuse large B-cell lymphoma.

**Fig.2:**
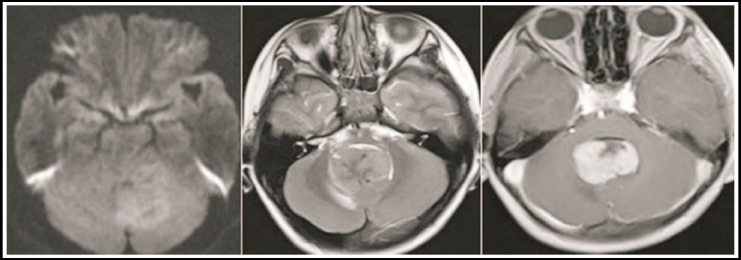
MRI image of 9-year-old Burkitt lymphoma.

**Fig.3:**
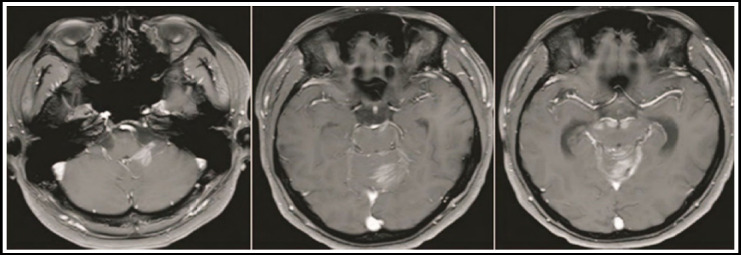
Burkitt lymphoma, a 29-year-old patient.

Of the 15 patients, 10 were single lesions, which occurred only under the curtain (Fig.1, Fig.2), five were multiple lesions, of which four lesions were found on and under the curtain, and one multiple lesion was located only under the curtain (Fig.1). Fig.3), the total number of lesions is 25. The 25 lesions include 5 lesions above the curtain in multiple lesions, and the remaining 20 lesions below the curtain include 15 cerebellar hemispheres and cerebellar vermiform (Fig.1, Fig.3), two midbrains (Fig.3), and bridge cerebellum. There are two horn regions (Fig.3) and one in the fourth ventricle (Fig.2). 24 lesions were solid and one lesion was cystic. The morphology of the lesions varies in size, showing a round shape (Fig.2), irregular shapes (Fig.1), nodular shapes (Fig.3), and patchy shapes (Fig.1-3, Fig.2, 3). 22 lesions on T1WI showed low or slightly low signal, 3 lesions showed moderate signal; 20 lesions on T2WI showed slightly higher signal, three lesions showed moderate signal, and 2 lesions showed mixed slightly higher signal. Edema of varying degrees occurred around the 12 lesions. After the enhancement scan, the solid parts of the 25 lesions showed obvious enhancement. Typical “sharp angle sign” and “umbilical concave sign” were seen ineight8 lesions, and 17 masses and nodules were uniformly enhanced (Fig.2). (Fig.3), four stripe-like enhancements (Fig.2), three patch-like enhancements (Fig.3), and one ring-like enhancement.

### Note

The horizontal axis T1WI (Fig.1), T2WI (Fig.2), and enhanced T1WI (Fig.3) show low signal in the left cerebellar hemisphere sheet T1WI, slightly higher signal in T2WI, and the enhanced scan is markedly patchy and DWI enhanced. A slightly higher signal was uneven.

### Note

The plain scan axis T2WI (Fig.1) and enhanced T1WI (Fig.2) show a medium-signal mass in the fourth ventricle, a fissure-like low signal in the mass, and the enhancement scan showed uneven enhancement.

### Note

The first axial enhancement T1WI (Fig.1, Fig.2) shows the abnormal enhancement of the patchy and striped plaques of the left cerebellar hemisphere beside the four ventricles and the cerebellar vermiform. The second axial T1WI enhanced T1WI after 20 days (Fig.3) Shows that the cerebellar vermiform lesions were significantly enlarged, and multiple nodular intensive lesions were found in the midbrain.

### DWI and MRS performance

Ten patients underwent DWI. Five patients showed homogeneous high signal, two patients showed uneven high signal, and three patients showed moderate signal. The ADC picture showed equal or low signal. The ADC value of tumor parenchyma in 10 patients was (0.62±0.095)×10-3mm2/s, while the ADC value of contralateral brain parenchyma was (0.73±0.038)×10-3mm2/s, and the difference was statistically significant. (P <0.05). Four cases underwent MRS examination, two cases showed a Lip peak at 1.33 ppm, and the other two cases did not see a Lip peak.

### Differential diagnosis

Metastatic tumors: similarities can be single or multiple, often accompanied by significant enhancement. For enhanced MRI images, those with larger metastatic tumors often have necrosis in the center. On imaging, the tumor has a circular enhancement, which is nodular, and lymphoma is relatively rare. Glioma: Most gliomas have long T1 and long T2 signals, showing obvious infiltrative growth. Among them, oligodendroglioma may have calcification, while central nervous system lymphoma calcification is rare. The most malignant glioblastomas are more solitary, and the tumor occupying effect is more obvious, but the enhancement is more irregular, and the edema around the tumor is more severe. Meningiomas are mostly located near the skull base or convex meningeal attachment site, most of them are clear-shaped, round, with some features of adjacent axial tumors such as hyperplasia or destruction of the skull, white matter collapse signs, and rare in lymphomas. On imaging, meningiomas usually show obvious uniform enhancement, and some of them show “meningeal tail sign”. Brain abscess: Most of them have a history of fever, and the imaging is typically a ring-shaped enhancement of the abscess wall, which can be distinguished from lymphoma according to the history of infection and the age of the typical abscess formation.

Due to the dense composition of tumor cells in PCNSL, less cytoplasm, high cytoplasmic ratio, and small extracellular space in the tumor, the diffusion of water molecules in PCNSL is often limited.

## DISCUSSION

Experiments showed that the focus of this group of cases is mainly located in the cerebellar hemisphere, and a few are in the cerebellar vermis, the four ventricles and the brain stem. It is generally believed that the incidence of rare sites of PCNSL is more common in people with immunodeficiency, such as patients with AIDS or patients who have been taking immunosuppressants for a long time after organ transplantation.^7^-^9^ However, all patients in this group had no history of congenital immune dysfunction or acquired immune dysfunction. PCNSL is common with a single lesion, but it can also appear as multiple lesions.^10^-^12^ A single lesion in this group occurred in the fourth ventricle. It was a round, solid, unevenly enhanced, clear border, and the patient was a 6-year-old boy.^13^-^16^ Therefore, he was diagnosed with medulloblastoma before surgery. 5 cases in this group are multiple lesions, and only 1 case has all the lesions located under the curtain, distributed in the cerebellar hemisphere, cerebellar vermiform part and midbrain, and strengthened in the form of patches at an early stage after enhancement. It is ineffective after hormone therapy, and the disease progresses rapidly. After multiple MRI reexaminations, the number of lesions increases, the volume increases, and the enhancement methods are different. They are nodular, striped, and sheet-shaped, but all are confined to the scene.

In this group of patients, mass-type PCNSL showed high or homogeneous signals on DWI, while non-massive lesions were mostly moderate signals. This group of 10 patients underwent a DWI scan. The measured ADC value of the parenchymal part of the tumor was (0.62±0.095)×10-3mm2/s, which was statistically different from the ADC value of the contralateral normal brain parenchyma. The PCNSL in the posterior cranial fossa needs to be distinguished from other subcancerous tumors. This study was a retrospective analysis. The equipment used in most cases was backward, and there was a lack of comparative studies on magnetic resonance spectroscopy (MRS), perfusion imaging (PWI), and diffusion imaging (DWI), etc. These functional imaging methods play an important role in the diagnosis and differential diagnosis of PCNSL. Therefore, further analysis should be made from these aspects in future studies. Differences in MRI images of complications of PCNSL were found, but not confirmed in terms of treatment. Only after large data analysis can its clinical application value be proved, so it will be further studied from this aspect.

## CONCLUSIONS

Although PCNSL is rare in the posterior cranial fossa, it still has certain characteristics. The predominant sites are the cerebellar hemisphere and cerebellar vermiform. After enhancement, in addition to the typical “acuminate sign” and “umbilical concavity sign”, it can also show nodular, striped, and patchy enhancement. DWI, ADC value and MRS can help improve the correct diagnosis rate of PCNSL. However, there are still many clinical manifestations of PCNSL in the posterior cranial fossa. The imaging findings are atypical, and early diagnosis is extremely difficult. Ultimately, they need to be confirmed by stereotactic biopsy or surgical pathology.

### Authors Contribution:

**GL:** Conceived the study, literature review, participated in its design, coordination, analyzed the data, helped to draft the manuscript and also the responsible and accountable for the accuracy or integrity of the work. **YL & XL:** Helped in design, data collection, article drafting & critical revision. **ZZ:** Takes the responsibility and is accountable for all aspects of the work in ensuring that questions related to the accuracy or integrity of any part of the work are appropriately investigated and resolved.
